# Altered brain network centrality in patients with mild cognitive impairment: an fMRI study using a voxel-wise degree centrality approach

**DOI:** 10.18632/aging.203105

**Published:** 2021-06-09

**Authors:** Jing Xiong, Chao Yu, Ting Su, Qian-Min Ge, Wen-Qing Shi, Li-Ying Tang, Hui-Ye Shu, Yi-Cong Pan, Rong-Bin Liang, Qiu-Yu Li, Yi Shao

**Affiliations:** 1 Department of Ophthalmology and Geriatric Medicine, The First Affiliated Hospital of Nanchang University, Jiangxi Center of Natural Ocular Disease Clinical Research Center, Nanchang 330006, Jiangxi, China; 2 Eye Institute of Xiamen University, School of Medicine, Xiamen University, Department of Ophthalmology, Zhongshan Hospital, Xiamen University, Xiamen, China; 3 Department of Ophthalmology, Massachusetts Eye and Ear, Harvard Medical School, Boston, MA 02114, USA

**Keywords:** degree centrality, Alzheimer's disease, mild cognitive impairment, functional connectivity, MMSE

## Abstract

Purpose: Previous studies in patients with Alzheimer’s disease have shown amyloid beta accumulation in the brain and abnormal brain activity, with mild cognitive impairment (MCI) in early stages of the disease. The aim of the current study was to investigate functional connectivity in patients with MCI.

Methods: We recruited 24 subjects in total, including 12 patients with MCI (6 men and 6 women) and 12 healthy controls (HCs) (6 men and 6 women), matched for age, gender, and lifestyle factors. All subjects underwent resting-state functional magnetic resonance imaging scans and voxel-wise degree centrality (DC) was used to evaluate alterations in the strength of brain network connectivity.

Results: The DC value of the left inferior temporal gyrus was lower in MCI but significantly higher in the right fusiform gyrus and the left supplementary motor area, compared with HCs. The DC value in left inferior temporal gyrus correlated positively with disease duration and negatively with Mini-Mental State Examination. ROC curve analysis of brain regions showed acceptable specificity and accuracy of DC values between MCIs and HCs in the area under the curve (right fusiform gyrus, 0.955; left supplementary motor area, 0.992; left inferior temporal gyrus, 1.000).

Conclusions: Abnormal functional connectivity in brain regions of patients with MCI may reflect the pathological process of Alzheimer’s disease development and could prove useful in clinical diagnosis and treatment.

## INTRODUCTION

Alzheimer’s disease (AD), a progressive and irreversible neurological disorder, is characterized by memory loss, aphasia, agnosia, visual and spatial skill impairment, executive dysfunction, and personality and behavioral changes. For these reasons, it is a common cause of dementia. It has a long preclinical period with pathophysiological changes occurring 15 to 20 years before the onset of symptoms [1, 2]. The prevalence of AD increases exponentially with age, as reflected by its frequent occurrence in the elderly [2]. No effective treatment is available for AD and late interventions often result in treatment failure. Early detection and diagnosis of AD require efficient humoral and imaging markers, which are still in the developing stage. Medical imaging, including diffusion tensor imaging, fluorodeoxyglucose positron emission tomography, magnetic resonance imaging (MRI), and other methods can detect brain lesions in patients with AD. Monitoring of neurodegenerative changes in the central nervous system can provide direct information on disease progression [3]. The early stage of AD involves damage to the hippocampus and entorhinal cortex, whereas diffuse brain atrophy is associated with the late stage of AD [4].

Mild cognitive impairment (MCI) refers to a minor but noticeable decline in cognitive abilities. It is a heterogeneous clinical entity with multiple causes. The European Consortium on Alzheimer’s Disease Working Group on MCI describes a neuroimaging- and biomarker-based three-step process to identify MCI patients with incipient or prodromal AD [5, 6]: 1) decline in cognitive functions during the past year, 2) although patients’ clinical assessment shows cognitive impairment, they do not suffer from dementia and no major impact on daily life, and 3) identification of MCI subtypes responsible for AD.

In addition, amyloid (Aβ) accumulation in the brain of early-stage AD patients with MCI and no other clinical symptoms assist in the identification.

Although functional magnetic resonance imaging (fMRI) is an expensive and time-consuming brain-imaging method, it objectively assesses brain functions.

For instance, voxel-wise degree centrality (DC), an fMRI-based method, provides major insights into the functional connectivity of the whole brain and has been widely used to assess the pathophysiological mechanism of several diseases [7]. In contrast with other methods, such as amplitude of low-frequency fluctuations (ALFF) [8] and regional homogeneity (ReHo) [9], DC does not involve defining regions of interest (ROI) and evaluate the connection strength of the whole human brain at the voxel level [10]. We used DC to evaluate changes in brain functional connectivity in patients with MCI and any relationship between these changes and clinical symptoms.

## MATERIALS AND METHODS

### Patients

We recruited 24 age-, gender-, and lifestyle-matched subjects into our study. These included 12 patients (6 males and 6 females) and 12 healthy controls (6 males and 6 females) from the First Affiliated Hospital of Nanchang University, China. All participants underwent resting-state fMRI. Voxel-wise DC was used to evaluate alterations in the strength of brain network connectivity.

### Methods

**Figure 1:**
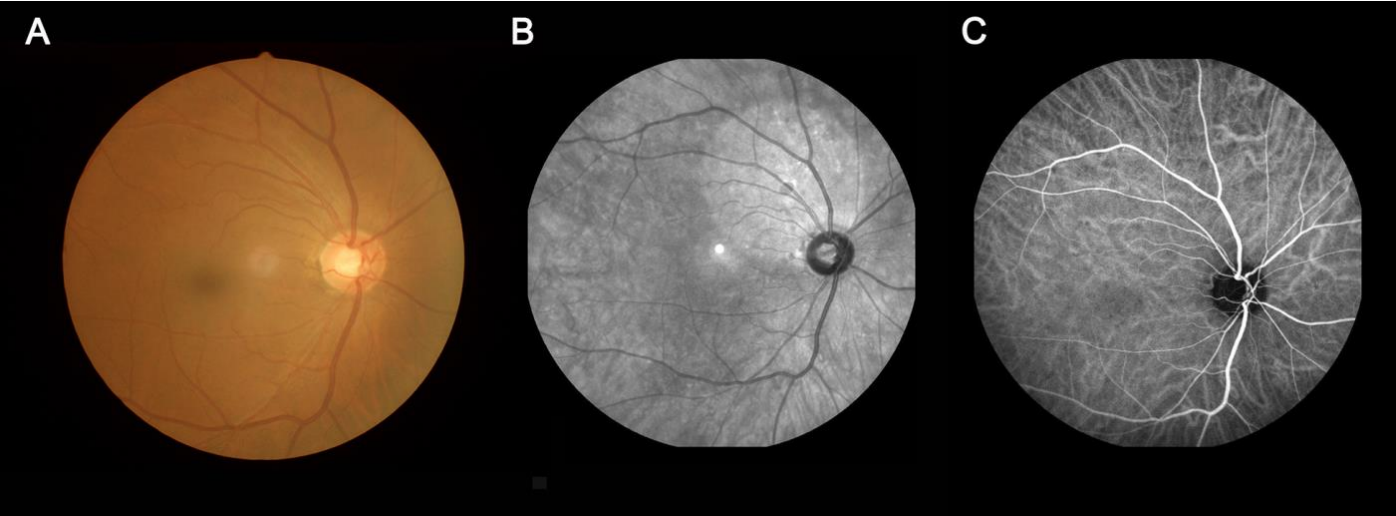
**An example of MCI.** (**A**) fundus photography; (**B**) fundus fluorescein angiography; (**C**) indocyanine green angiography. Abbreviations: MCI, mild cognitive impairment.

The eye fundus was examined using ophthalmic fundus photography, indocyanine green angiography (ICGA), and fundus fluorescein angiography (FFA) (Figure 1). The inclusion criteria were (1) age ≥ 45 years, (2) complaints of memory loss confirmed by the informant, and Mini-Mental State Examination (MMSE) score < 27, (3) activities of daily living scale (Barthel Index) ≥ 90, and (4) cranial MRI performed in the past 6 months not revealing parenchymal brain lesions.

The inclusion criteria of healthy controls (matched with the MCI group for gender and activities of daily living) were: (1) age ≥ 45 years, (2) routine brain MRI showing no significant abnormalities, (3) normal memory with an MMSE score ≥ 27, (4) no neurological diseases, mental illnesses, or cardiovascular diseases, (5) no drug or alcohol addiction, and (6) able to undergo MRI examination.

In addition, patients with other types of dementia, such as vascular dementia, Parkinson’s disease dementia (PDD), frontotemporal lobar degeneration, as well as cognitive impairment caused by other causes (such as drugs, alcohol dependence, tumors, epilepsy, and hydrocephalus), acute cerebral hemorrhage, cerebral infarction or intracranial space-occupying lesions, were excluded from the study. Patients with other mental illnesses, such as severe affective disorders or current evidence of depression, and those with vision, hearing impairment, or severe dementia were also excluded.

The research methods were consistent with the principles of the Declaration of Helsinki. All subjects volunteered to participate and were informed in advance about the aims, methods, and potential risks of the study. This study was approved by the Ophthalmic Medical Ethics Committee of the First Affiliated Hospital of Nanchang University.

### MRI data collection

All MRI data were recorded using a SiemensTrio 3.0 T scanner by implementing an 8-channel phased-array head coil in the First Affiliated Hospital of Nanchang University, China. All subjects underwent MRI scanning using parameters reported previously [11].

### Resting-state fMRI data preprocessing

All functional data were pre-filtered using the MRIcro program (https://www.MRIcro.com). The data were processed using SPM8 (http://www.fil.ion.ucl.ac.uk/spm), DPARSFA (http://rfmri.org/DPARSF), and a resting-state data analysis toolkit (http://restfmri.net/forum/index.php). The remaining 230 volumes collected from each patient were corrected for differences in slice acquisition time. The resultant images were subsequently realigned to correct for small movements between the scans. Patients with a maximum displacement of more than 3 mm in any direction (x, y, or z) or an angular rotation of more than 3 degrees in any of the 230 volumes were excluded from the study based on recorded kinematic error correction estimates (one patient). Differences in DC values between MCI and healthy control (HC) patients were compared (P < 0.05, Gaussian random field theory). More details of data-processing methods used have been described previously [8].

### Degree centrality

DC values were calculated based on the functional network of individual voxels by calculating the binarized adjacency matrix degree or threshold correlation between the participants as follows [7]:

Z_i_ = DC_i_–mean (DC of all cortical voxels)/standard deviation (DC of all cortical voxels)

### Brain–behavior correlation analysis

All subjects’ clinical data were collected to study any relationship between these and mean DC values of different brain areas.

### Statistical analysis

GraphPad Prism Version 9.0 was used for statistical analysis. An independent samples *t*-test was performed to evaluate different clinical manifestations between MCI and HC groups. Correlation analysis (using unpaired Student’s *t*-tests) was used to analyze the relationship between mean DC value and performance data collected from subjects’ records. P-values less than 0.05 were considered significant. Receiver operating characteristic (ROC) curves were used to assess DC values as a diagnostic marker in specific brain areas. Diagnostic accuracy was indicated by the area under the curve (AUC), with values between 0.5 and 0.7, 0.7 and 0.9, and >0.9 having low, moderate, and high accuracy, respectively.

## RESULTS

### Demographics

The two groups did not show any differences in age (P = 0.595) and gender (P > 0.99). The mean duration of MCI was 12.00 ± 15.42 months, which was different between HC and MCI groups (P < 0.001). In addition, S100β showed a remarkable difference (P < 0.001) (more details are provided in Table 1).

**Table 1:** Demographic characteristic of the enrolled subjects.

**Condition**	**MCI**	**HC**	**t**	**P**
**Subject**	12	12	NA	>0.99
**Age (y)**	64.33±7.01	64.00±6.18	0.124	0.595
**Gender (M:F)**	6:6	6:6	NA	>0.99
**Duration (month)**	12.00±15.42	NA	4.766	<0.001
**SBP**	128.92±12.00	130.5±10.83	-0.339	0.593
**DBP**	76.08±12.15	76.17±10.57	-0.018	0.593
**HR**	70.01±9.16	70.42±8.36	-0.091	0.922
**Barthel Index**	99.58±1.44	100	/	/
**MMSE**	21.42±4.56	27.83±2.52	-4.266	0.125
**Best-corrected VA-left eye**	0.29±0.10	0.23±0.06	1.967	0.164
**Best-corrected VA-right eye**	0.28±0.12	0.21±0.08	1.591	0.078
**S100β**	0.18±0.09	2.71±1.05	-8.327	<0.001

*P <0.05 Independent t-tests comparing two groups.

Abbreviations: MCI, mild cognitive impairment; HC, Healthy control; NA, not applicable; SBP, systolic blood pressure; DBP, diastolic blood pressure; HR, heart rate; MMSE, Mini-Mental State Examination; VA, visual acuity.

### DC comparison between groups

**Figure 2:**
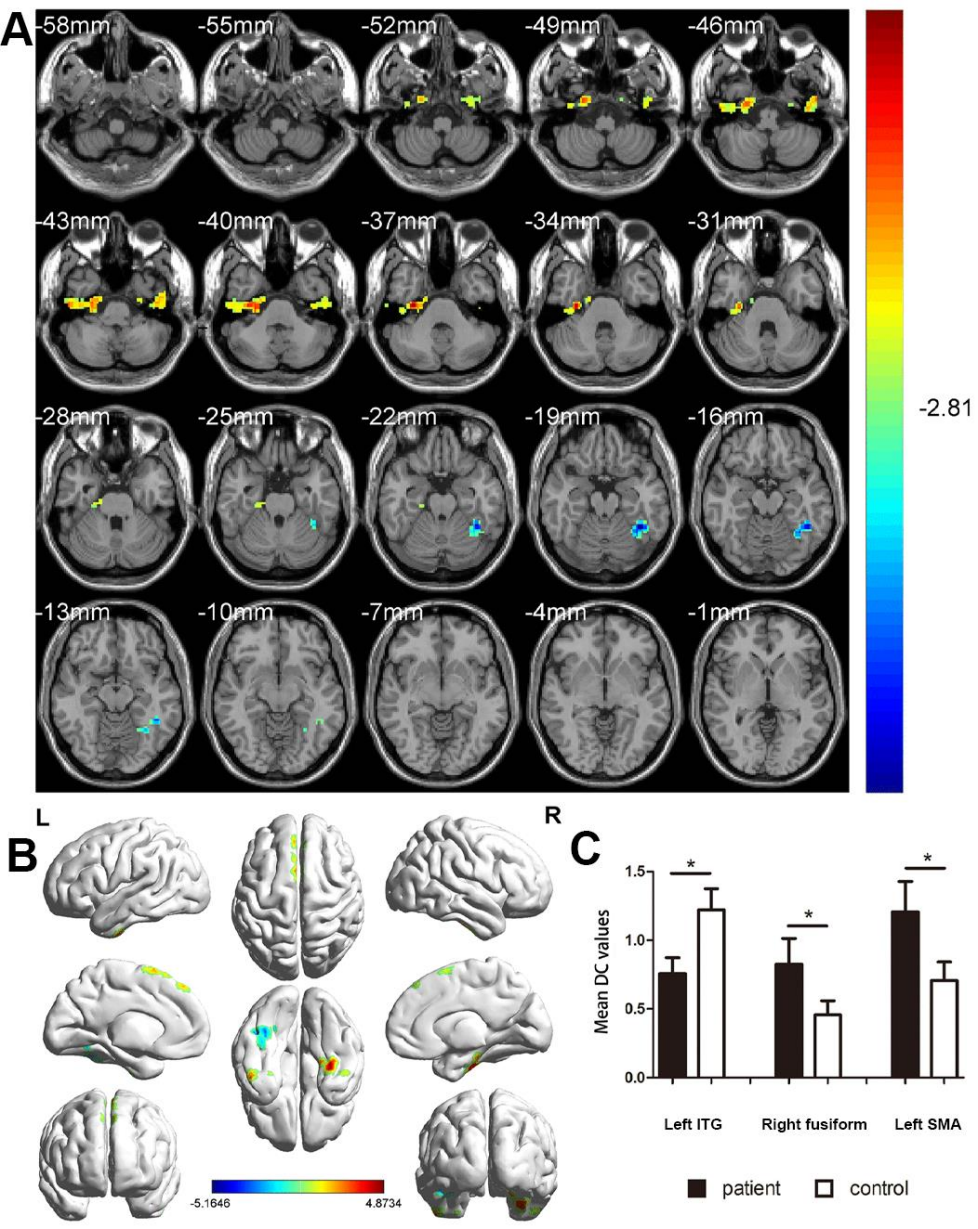
**Comparison of DC values in MCI and HC groups.** (**A**) Differences in DC were found in left ITG, right fusiform gyrus, and left SMA. (**B**) The stereoscopic form of the cerebrum. The red area indicates an increase in DC value; the blue indicates a decrease in DC value. (GRF correction, the cluster-level: P<0.05; two-tailed, with voxel level P<0.005). (**C**) The Mean DC value between MCIs and control group. Abbreviations: DC, Degree centrality; MCI, mild cognitive impairment; HC, healthy controls; ITG, inferior temporal gyrus; SMA, supplementary motor area.

**Figure 3:**
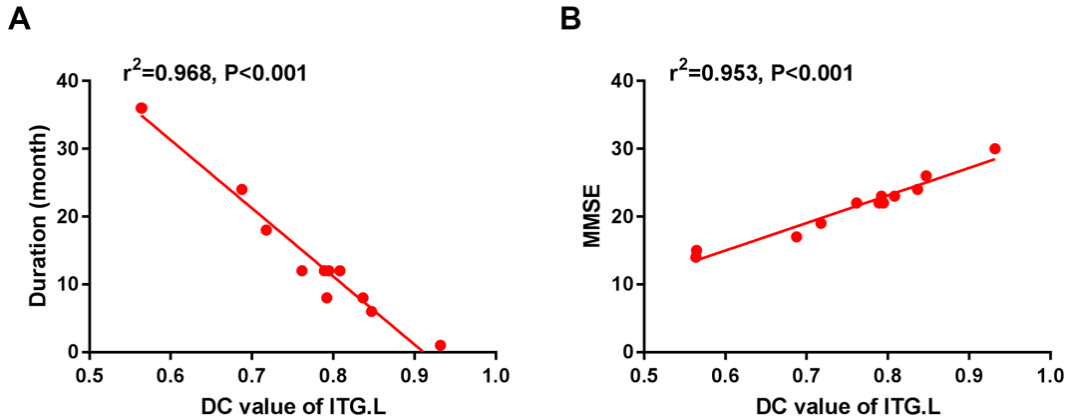
The correlation between the mean DC value of left ITG and the duration (**A**) and MMSE (**B**). In the Alzheimer's disease group, the mean DC value of left ITG showed a negative correlation with duration (r^2^=0.968, P<0.001). The mean DC value of the left ITG was positively correlated with MMSE (r^2^=0.953, P<0.001). Abbreviations: DC, Degree centrality; MCI, mild cognitive impairment; ITG, inferior temporal gyrus; L, left; MMSE, Mini-Mental State Examination.

The DC value was higher in MCI patients than in HC patients in the right fusiform gyrus and left supplementary motor area (SMA), whereas it was lower than that in HC patients in the left inferior temporal gyrus (ITG) (Figure 2 and Table 2). In the MCI group, the DC value in the left ITG was negatively correlated with disease duration (r^2^ = 0.968, P < 0.001, Figure 3A) and positively with MMSE score (r^2^ = 0.953, P < 0.001, Figure 3B).

**Table 2:** Brain areas with different DC values between MCIs and HCs.

**Brain areas**	**MNI coordinates**			**BA**	**Number of voxels**	**T value**
	**X**	**Y**	**Z**			
HCs>MCIs						
Left ITG	-42	-48	-18	55	110	-5.1646
HCs<MCIs						
Right fusiform	33	-18	-36	-	260	4.6357
Left SMA	3	42	57	-	119	4.8734

Statistical thresholds were set at voxel (P < 0.01) and multiple comparisons were performed using GRF theory (z > 2.3, cluster P < 0.05 corrected).

Abbreviations: DC, degree centrality; MCI, mild cognitive impairment; HC, healthy control; MNI, Montreal Neurological Institute; BA, Brodmann area; SMA, supplementary motor area; ITG, inferior temporal gyrus.

### ROC curve

**Figure 4:**
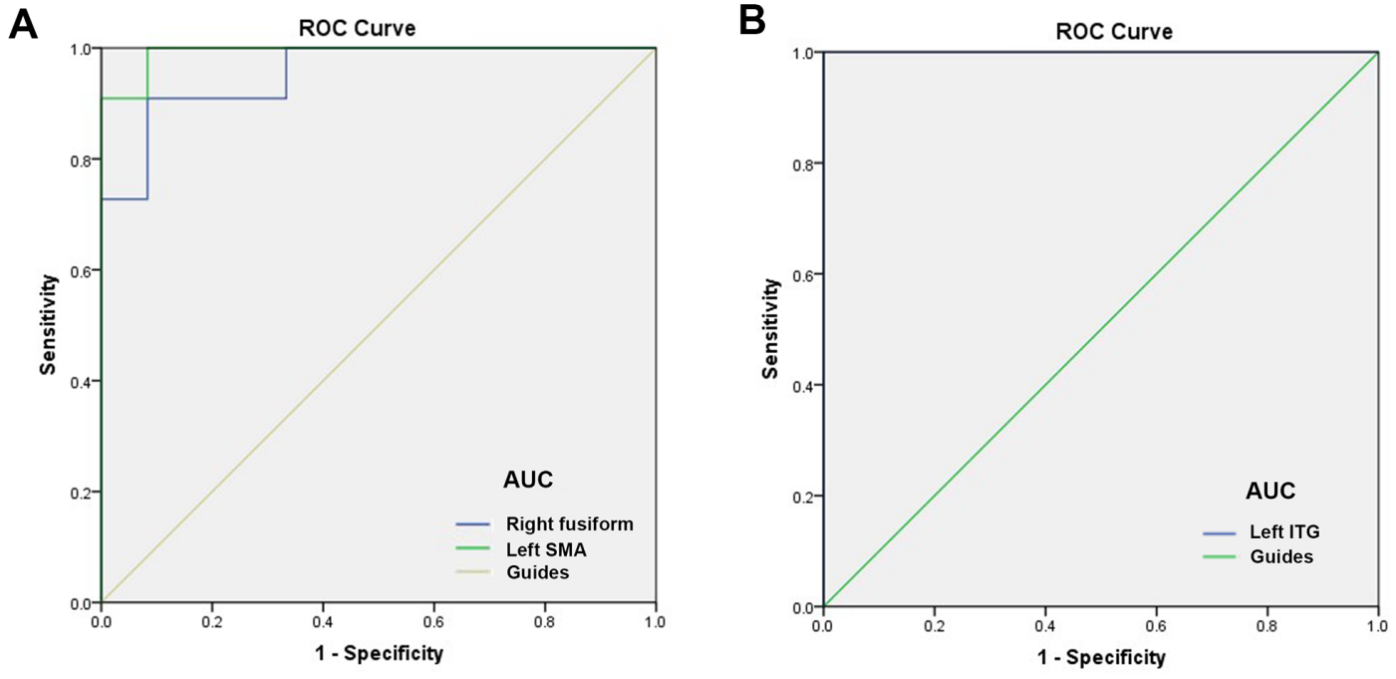
**ROC curve analysis of the mean DC values for altered brain regions.** (**A**) The AUC were 0.955, (*p*<0.0001; 95% CI: 0.877-1.000) for right fusiform, and left SMA 0.992, (*p*<0.0001; 95% CI: 0.968-1.000). (**B**) The AUC of left ITG were 1.000, (*p*<0.0001; 95% CI: 1.000-1.000). Abbreviations: ROC, receiver operating characteristic; AUC, area under the curve;ITG, inferior temporal gyrus; SMA, supplementary motor area.

The ROC curve was used to analyze the mean DC values of patients with MCI and HC. The larger the area under the curve (AUC), the higher was the diagnostic value. AUC values were right fusiform gyrus, 0.955; left SMA, 0.992; and left ITG, 1.000 (Figure 4A, MCIs > HCs; Figure 4B, MCIs < HCs).

## DISCUSSION

**Figure 5:**
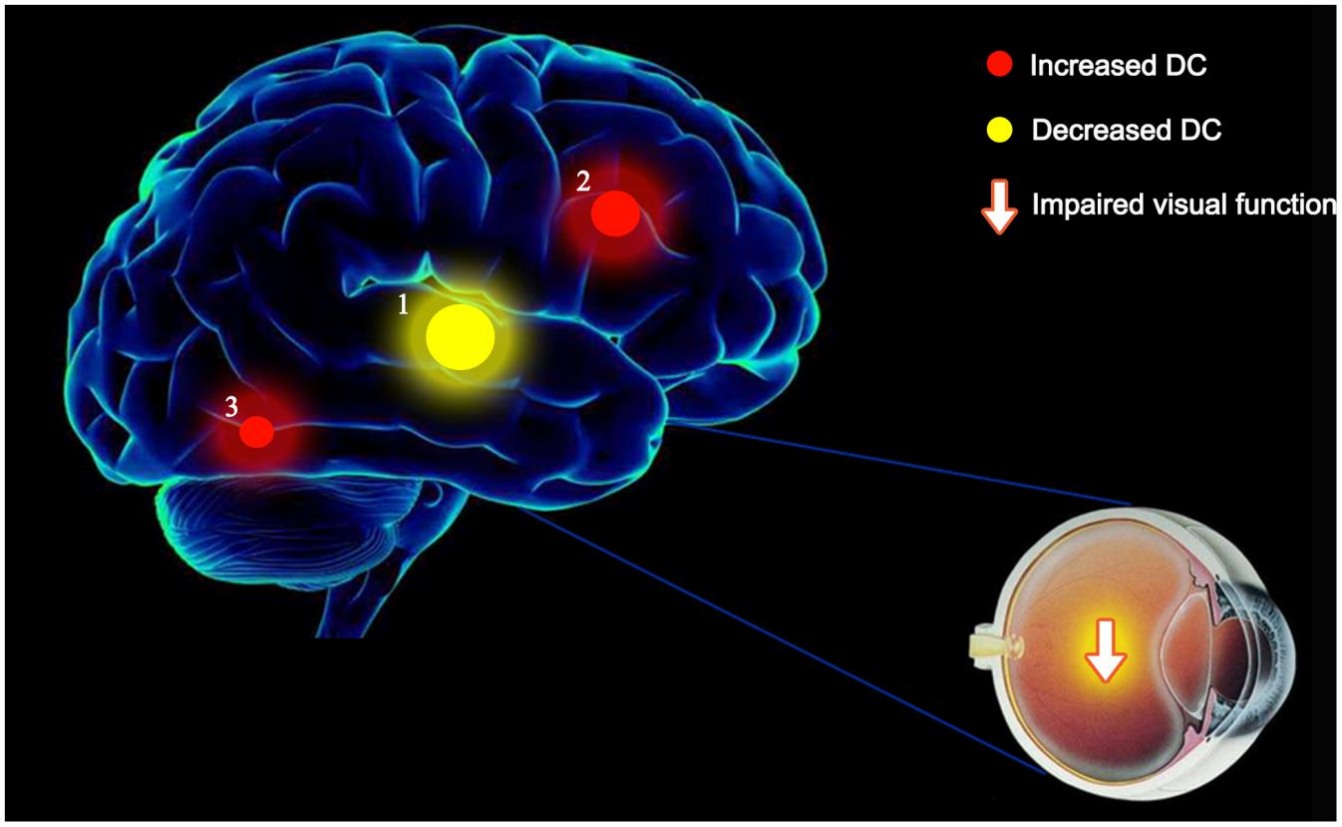
**The mean DC values of altered brain regions.** Compared with the HCs, the DC values of the following regions were decreased to various extents: 1- left ITG (BA 55, t = -5.1646). Compared with the HCs, the DC values of the following regions were increased to various extents: 2- left SMA (t = 4.68734), 3- right fusiform (t = 4.6357). Abbreviations: DC, Degree centrality; HCs, healthy controls; BA, Brodmann's area;ITG, inferior temporal gyrus; SMA, supplementary motor area.

**Figure 6:**
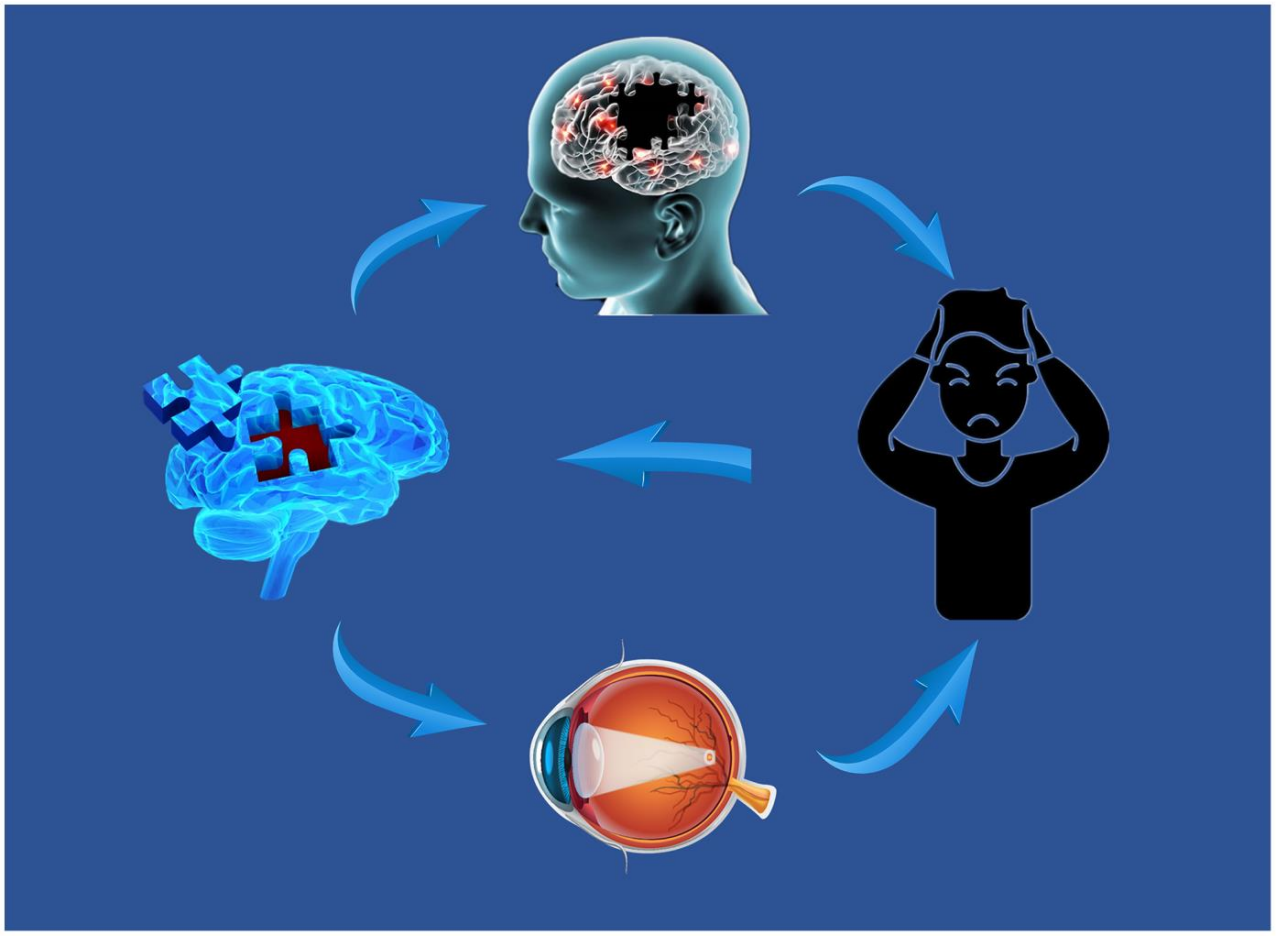
**Relationship between MRI images and clinical manifestations in MCI.** Abbreviations: MRI, magnetic resonance; MCI, mild cognitive impairment.

DC is a reliable, resting-state fMRI method to measure brain activity and connectivity. It has been successfully applied to study several neurogenic and ophthalmological diseases (Table 3). While assessing the brain activity in patients with MCI, we found the DC values to be lower than those in controls in the left ITG but relatively high in the left SMA and right fusiform gyrus. Furthermore, this alteration occurred earlier than the changes in the eyes (Figure 5). In addition, the left ITG showed a positive correlation with MMSE in MCI patients and a negative correlation with disease duration (Figure 6).

**Table 3:** Brain areas alteration and its potential impact.

**Brain areas**	**Experimental result**	**Function**
Inferior temporal gyrus	HCs>MCIs	Related to cognitive learning and object memory, emotional processing
Fusiform gyrus	HCs<MCIs	Face recognition, Secondary classification and recognition of objects
Supplementary motor area	HCs<MCIs	Language expression, movement; transforming emotional experiences into movement

Abbreviations: HC, healthy control; MCI, mild cognitive impairment.

The temporal lobe is located below the lateral fissure and processes vision, olfaction, sensation, and memory. The relatively low DC values in the left ITG in MCI patients suggest impaired MCI. We used the available data to study the association between MMSE and disease duration. The DC values in the left ITG decreased with duration (Figure 3A) but were positively correlated with MMSE scores (Figure 3B). These results show that MCI develops over time and affects the cognitive process.

In addition, the data revealed higher Sβ100 in the MCI group than in the HC group. Previous studies have reported this phenomenon in AD [12] but not in MCI. Thus, high S1β00 is related to the development of AD and could be a potential biomarker to identify MCI subtypes leading to AD.

Abnormal phosphorylation of tau protein hinders cell–cell connections, disrupts the axons of neurons, and causes pathological damage to the body. Accumulation of Aβ greatly accelerates the normal spread of tau with aging through neuronal communication pathways [13]. This phenomenon is observed in patients with AD and manifested as plaques formed by β-amyloid and tangles of neurofibrils within nerves, severely impairing brain function. In addition, it is speculated that plaque accumulation initiates during MCI development, causing low DC values of left ITG. The temporal lobe, from which these lesions emanate [14–17], coordinates social, emotional, language functions, and long-term episodic memory and is associated with autism and language deficits [18]. Dehaene et al. [19] proposed the concept of a “local combined detector” and suggested the function of ITG in information transmission and language learning [20].

In addition, ITG is involved in common object perception. A study [21] found reduced ITG and fusiform gyrus function in autistic patients during face recognition, a finding consistent with that of our study. However, the present results are inconsistent with those reported previously [22]. We attribute these inconsistencies to differences in age between patients in different studies.

Aβ accumulates in the retinas of patients with MCI, probably before accumulating in the brain [23]. In addition, cataract patients are 1.43 times more likely to have AD than normal individuals [24]. Although we did not study whether the vision was impaired in patients with MCI, AD can be complicated by ocular diseases, causing visual impairment [25]. Thus, Aβ in the eyes can serve as an early clinical indicator for AD prior to clinical symptoms.

Fusiform gyrus, a region in the occipitotemporal lobe, participates in advanced visual processing, especially target recognition and categorization of complex stimuli (such as human faces). People with autism have cognitive defects associated with the fusiform gyrus, including impaired facial processing and abnormalities in the fusiform gyrus. Patients with MCI are at an increased risk of depression and anxiety [26, 27]. Although the enhanced activity of the right fusiform gyrus has been found in patients with depression, a study showed it not be associated with these symptoms [28]. We suspect that increased DC values observed in the right fusiform gyrus in MCI patients compared to HC patients could be potentially associated with the occurrence of depressive symptoms.

SMA is located on the medial side of the primary motor cortex and the medial part of the premotor cortex, roughly equivalent to Brodmann’s area 6 (BA6). It is composed of two regions, pre-SMA (rostral side) and SMA proper (caudal part). Although SMA is more active than the cerebral cortex during simple movements such as limb movements, it is associated with complex motor memory as well. It coordinates the temporal organization of movements, especially the execution sequence of multiple movements [29]. In addition, SMA proper is implicated in the planning of complex movements of learning (believed to be internally driven than by visual cues) and the coordination of movements involving both hands. Pre-SMA participates in the learning of new motion sequences. A previous study reported increased interhemispheric functional connectivity with Aβ accumulation in SMA. In addition, we reported a higher DC value of SMA in the MCI group than in the HC group. These findings suggest a compensatory enhancement through the recruitment of other networks for non–amyloid-dominant dementia [12].

This study has certain limitations. First, the number of patients was limited, resulting in low reproducibility of results and overestimation of effects. Second, inevitable individual differences could have impacted the experimental results. Finally, patients’ daily lifestyles will inevitably affect the development of MCI.

## CONCLUSIONS

MCI can lead to anomalies in specific regions in the patient’s brain and induce a variety of symptoms, such as affective expression disorders and retinopathy. These findings preliminary validate the relevant theories, such as brain abnormalities due to Aβ accumulation in AD, and provide a basis to further investigate neural changes in MCI and AD. We believe these findings will assist in the early diagnosis of MCI and predict the development of MCI into AD.

### Ethical Statement

All research methods were approved by the committee of the medical ethics of the First Affiliated Hospital of Nanchang University and were in accordance with the 1964 Helsinki declaration and its later amendments or comparable ethical standards. All subjects were explained the purpose, method, potential risks and signed an informed consent form.
